# Application of Patient-Derived Cancer Organoids to Personalized Medicine

**DOI:** 10.3390/jpm12050789

**Published:** 2022-05-13

**Authors:** Masahiro Shiihara, Toru Furukawa

**Affiliations:** Department of Investigative Pathology, Tohoku University Graduate School of Medicine, Sendai 980-8575, Japan; shiihara.masahiro_1@twmu.ac.jp

**Keywords:** personalized medicine, precision medicine, organoid, cancer, patient-derived cancer model

## Abstract

Cell models are indispensable for the research and development of cancer therapies. Cancer medications have evolved with the establishment of various cell models. Patient-derived cell lines are very useful for identifying characteristic phenotypes and susceptibilities to anticancer drugs as well as molecularly targeted therapies for tumors. However, conventional 2-dimensional (2D) cell cultures have several drawbacks in terms of engraftment rate and phenotypic changes during culture. The organoid is a recently developed in vitro model with cultured cells that form a three-dimensional structure in the extracellular matrix. Organoids have the capacity to self-renew and can organize themselves to resemble the original organ or tumor in terms of both structure and function. Patient-derived cancer organoids are more suitable for the investigation of cancer biology and clinical medicine than conventional 2D cell lines or patient-derived xenografts. With recent advances in genetic analysis technology, the genetic information of various tumors has been clarified, and personalized medicine based on genetic information has become clinically available. Here, we have reviewed the recent advances in the development and application of patient-derived cancer organoids in cancer biology studies and personalized medicine. We have focused on the potential of organoids as a platform for the identification and development of novel targeted medicines for pancreatobiliary cancer, which is the most intractable cancer.

## 1. Introduction

Cancer is a major threat to human health. With the development of next-generation sequencing technology, the genetic information of various cancers has gradually been clarified. Research and development of molecular-targeted drugs based on the genetic information of cancers have been conducted worldwide. However, only a few drugs have been approved by the Food and Drug Administration (FDA) after clinical trials [1,2]. Thus, although evolving, very few therapeutic options are available for some types of cancer. Nevertheless, in the near future, therapeutic agents will be selected based on genetic alterations rather than the location of the cancer, known as genome-based personalized medicine.

Cell models are indispensable for research and the development of cancer therapies. Cancer medication has evolved with the establishment of various cell models. Patient-derived cell lines are very useful for identifying characteristic phenotypes and susceptibilities to anticancer drugs, as well as molecular targeted therapies for patient tumors; however, conventional 2-dimensional (2D) cell cultures have several drawbacks in terms of engraftment rate and phenotypic changes during culture [3]. The organoid is a recently developed in vitro model with cultured cells that form a three-dimensional (3D) structure in the extracellular matrix. Organoids have the capacity to self-renew and can organize themselves to resemble the original organ or tumor in terms of both structure and function. Patient-derived cancer organoids (PDCOs) may be more suitable for cancer research than conventional 2D cell lines or patient-derived xenografts (PDXs).

Herein, we review the current status and future prospects of PDCOs. In particular, we focused on pancreatobiliary cancer organoids and their application in personalized medicine. Our aim was to present their potential as a cancer model to understand cancer biology and test therapeutics in vitro, which will lead to clinical applications and further translational research.

## 2. Three-Dimensional Culture

Mammalian cells have self-organizing abilities, not only in vivo but also in vitro. Even after complete dissociation, cells can re-aggregate and reconstruct the original architecture of their organs. Recently, this outstanding feature has been used to rebuild organ parts or even complete organs from tissues or embryonic stem cells, which are called organoids. Organoid culture is a 3D culture method that maintains tissue-specific functions in vitro. Reports of 3D cultured cells were published more than 30 years ago. Li et al. reported in 1987 that when mammary epithelial cells were cultured in an extracellular matrix, they succeeded in culturing a small conduit with a 3D mammary structure [4]. In the same year, Shannon et al. succeeded in creating a 3D type 2 alveolar cell culture in a similar way [5]. These 3D cell cultures are considered prototypes of organoids.

## 3. Organoid Culture

Organoids are classified into two types according to their stem cell characteristics. The first is organoids derived from pluripotent stem cells, and the other is organoids derived from adult stem cells [6,7,8]. The latter is discussed in this review. Organoids can be obtained from both isolated adult stem cells and tissue fragments from the corresponding organ. Under appropriate conditions, cells can proliferate in culture for a long time while maintaining their genetic stability and commitment to their tissue of origin. These cultures can serve for studying stem cell biology as models of adult functional tissues and somatic mutational processes. Organoids have a self-organizing ability in a three-dimensional structure with differentiated epithelial cells, revealing key functions as mini-organs under the control of growth factors and suppressors added to the medium. With the identification of various niche factors that maintain specific microenvironments, it has become possible to culture organoids derived from various organs. These niche factors include Wnt, epidermal growth factor (EGF), fibroblast growth factor, extracellular antagonist of BMP proteins (Noggin), inhibitor of TGF-β type I receptor (A83-01), prostaglandin E2, nicotinamide, R-spondin, gastrin-I, and N-acetylcysteine amide [9,10,11,12,13,14,15,16,17,18,19]. Organoids derived from various organs with adapted protocols have been reported, including the mammary gland [20], bone [21], small intestine [22,23], stomach [15,24], colon [13,14,25], lung [26], liver [27] [16,28], pancreas [16,29], prostate [18,30], fallopian tube [17], salivary gland [31], and tongue [32]. Notably, organoid technologies are versatile. Theoretically, organoid cultures can be initiated from small tissue samples, typically obtained by biopsy or from surgical specimens. Generally, no stem cell purification is necessary, and seeding of small, enzymatically disrupted tissue fragments results in the rapid sealing of epithelial fragments to form cystic structures lined by polarized epithelial cells.

## 4. Cancer Organoid

**Figure 1:**
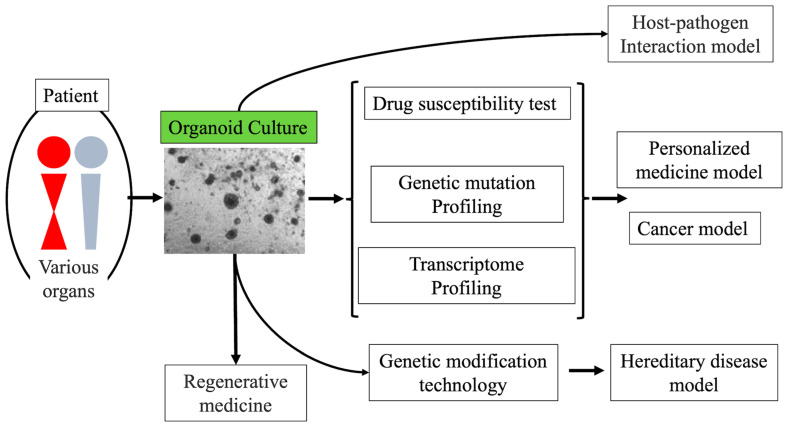
Applications of patient-derived organoids. Organoids have various potential applications in regenerative medicine, host-pathogen interaction models, hereditary disease models, and cancer models.

Organoids have great potential for use in various applications, such as genetic diseases, immunotherapy, and cancer progression models [33,34] (Figure 1). Among them, the published literature indicates the superiority of organoids as cancer models over 2D cell lines or PDXs. Although cancer 2D cell lines and PDXs have contributed significantly to cancer research as commonly used human cancer models, they have some disadvantages. First, the success rates of establishing cell lines are generally low, i.e., the establishment of cancer cell lines from primary tissue is very inefficient, mainly because of the challenge for cells to adapt to in vitro 2D culture conditions. Furthermore, only a few clones are supposed to survive and expand, and therefore, sometimes the cultures do not faithfully recapitulate the genetic spectrum of the original tumor. Thus, 2D cell lines may not be more suitable than organoids for studying the driver mutations required for tumor initiation and progression [3]. PDXs are generated by transplanting patient-derived fresh tissue into the subcutis or an orthotopic site in immunodeficient mice. Compared to 2D cultures, PDXs can recapitulate the tumor microenvironment in the 3D structure, including the interaction of cancer cells with stroma with blood vessel formation. However, PDX is a labor-intensive and high-cost method that has difficulty in engraftment and requires significant investments in resources for its maintenance, so it is poorly suited for high-throughput drug screening or genetic manipulation. Furthermore, this approach is time-consuming and may require a host (mouse)-specific tumor adaptation [35]. In recent decades, several human-derived cancer organoid cultures have been established, and some data utilizing the properties of organoids have been reported [16,29,36,37,38,39]. The success rate of PDCO cultures varies considerably depending on the type of tumor. The success rates for establishing organoid lines for colon, breast, lung, and liver cancers were reported to be 90%, >80%, 70%, and ~100%, respectively [36,40,41,42]. However, culture is quite difficult, and the success rates are still low for some kinds of tumors or early-stage tumors such as biliary tumors, intraductal papillary mucinous neoplasms of the pancreas, and ovarian tumors [36,37,43,44,45]. As detailed in later sections, cancer organoids have evolved into research that combines genomic editing techniques with drug susceptibility studies, immunotherapy, and tumor microenvironmental research [38,46].

## 5. General View of Pancreatobiliary Cancer

Pancreatobiliary cancer (PBC) is an intractable disease with a poor prognosis. The 5-year survival rate for pancreatic cancer is <8%, which is the worst among solid cancers. The 5-year survival rate for biliary cancer is approximately 25%, which is considerably lower than that for other gastrointestinal cancers. The incidence and mortality rates of PBC have been increasing worldwide over the past few decades. Curative resection with a negative surgical margin is necessary to prolong survival. Surgical resection remains the best treatment option; however, a high recurrence rate after surgery significantly affects disease outcomes [47,48,49]. However, many patients present with unresectable tumors at the time of PBC diagnosis, and they have limited chemotherapeutic options [50,51]. Therefore, there is an urgent need to develop effective therapeutic agents. The genetic background of PBC has been clarified with the development of next-generation sequencing technologies. In recent years, a number of molecular-targeted drugs have been developed, some of which have been used in PBC patients. For example, in biliary cancer, progression-free survival was significantly improved with ivosidenib, an orally available inhibitor of isocitrate dehydrogenase type 1 (IDH1) [52]. Furthermore, the efficacy of pre- and post-chemotherapy for PBC has been tested [51,53]. In pancreatic cancer, adjuvant chemotherapy has become the new standard of care for resected pancreatic cancer; further, neoadjuvant chemotherapy could also be a standard protocol [51,53]. The treatment of PBC has shifted from an era of extended surgery to an era of multimodal therapy.

PBCs have distinctive mutational profiles compared with other cancers. Pancreatic cancers frequently harbor mutations in *KRAS, CDKN2A, TP53*, and *SMAD4* [54,55]. In particular, more than 90% of pancreatic ductal adenocarcinomas have *KRAS* mutations, which indicates the essential incorporation of aberrations in the RAS signaling pathway in pancreatic cancer. Moreover, multiple pathways and processes other than RAS signalings, such as CDK, TGF-β, SHH, JNK, and integrin signaling, are complicatedly altered in pancreatic cancers, in which some of the alterations often overlap [54,55]. Biliary cancers harbor mutations in diverse genes, including *ARID1A, ELF3, ERBB2, IDH1, IDH2, KRAS, PIK3CA*, and *TP53* [56]. There are no characteristic somatic mutations widely found in biliary cancers; therefore, mutations differ in each tumor. Somatic mutations in cancers often provide an opportunity for molecular-targeted therapy. However, the diversity of somatic mutations in individual patients necessitates the design of personalized therapeutic strategies.

## 6. Pancreatobiliary Cancer Organoid

There are fewer reports on patient-derived PBC organoids compared with other cancers. Although a protocol for culturing PBC organoids has been reported in recent years, the culture success rate is still lower than that of other cancers. Several factors may underlie the difficulty in culturing PBC organoids. First, resected specimens of PBC contain a considerable amount of stroma and relatively few viable tumor cells. This was especially obvious in pancreatic cancer samples obtained after neoadjuvant chemotherapy [43]. In our data, the culture success rate in the neoadjuvant chemotherapy group (2/10, 20.0%) was significantly lower than that in the non-neoadjuvant chemotherapy group (28/44, 63.6%; *p* = 0.016). Second, PBC has distinctive mutational profiles compared with other cancers, as mentioned in the previous section. These distinctive mutational profiles in PBC suggest that distinct culture conditions are required to grow organoids by adjusting growth factors to fit the mutational profile. We found that organoids cultured from the tumor parts of surgical specimens were a mixture of normal epithelial organoids and cancer organoids and experimentally showed that it was necessary to selectively remove the normal organoids to culture cancer organoids. Organoids derived from normal epithelial cells appeared in balloon-like shapes, which lacked mutations in the primary tumor, whereas organoids derived from cancer cells appeared in solid shapes, which indeed harbored mutations in the primary tumor [43]. Seino et al. reported a method for the selective culture of pancreatic cancer organoids by adjusting the growth factors added to the culture medium [57]. In their protocol, owing to the high prevalence of *KRAS* mutations in pancreatic ductal adenocarcinoma (PDAC), the organoids are first placed in an EGF-depleted condition to enrich cancer organoids with an autonomously active EGFR-RAS pathway. Organoids that are susceptible to EGF removal are alternatively treated with Nutlin3 (an MDM2 inhibitor) or Noggin removal/BMP4 to select potentially existing *TP53* or *SMAD4* mutant organoids, respectively [57].

As in biliary cancers, the mutations are not only distinctive from other cancers but also diverse among individual tumors, as demonstrated by us and others [43,56]. This means that appropriate culture conditions should differ depending on the tumor. We succeeded in culturing biliary cancer organoids with a high probability, including rare tumors such as intraductal papillary neoplasm of the bile duct of the extrahepatic bile duct, intracholecystic papillary neoplasm, and adenosquamous cell carcinoma of the gallbladder [43].

Heterogeneity in a tumor, such as intraductal papillary neoplasm of the bile duct, also matters and often causes extreme difficulties in culture [44]. This is because the tissue pieces collected for culture inherit only some of the mutations in the entire tumor. Even in genetically diverse biliary cancers, PDCO has been shown to maintain genetic diversity in long-term cultures under appropriate conditions [37].

There are two major objectives of recent studies of PBC organoids. The first is the screening of therapeutics using cancer organoids. The second is to explore cancer biology, such as the interactions between tumor cells and the tumor microenvironment. Driehuis et al. established a platform consisting of 27 patient-derived pancreatic cancer organoids that were used for high-throughput drug screening, with a panel of 76 different therapeutic agents [58]. Hu et al. identified SIRT5 as a key tumor suppressor in PDAC in therapeutic studies using organoids and PDXs [59]. Fujiwara et al. studied the distinct 5-ALA-based photodynamic activity on cholangiocarcinoma (CCA) organoids and suggested its diagnostic potential for the discrimination of CCA from non-tumor tissues [60].

PBC with abundant stroma may require research and treatment strategies targeting not only cancer cells but also the tumor microenvironment, including fibroblasts or immune components. Tsai et al. established a co-culturing system for pancreatic organoids, fibroblasts, and T cells. They visualized T cells at the boundary of Matrigel domes containing organoids infiltrating into the Matrigel, migrating toward the organoids, and diffusing the boundary [61]. Koikawa et al. compared the conditions between direct contact and indirect contact co-culture systems of organoids and pancreatic stellate cells (PSCs). Their results implied that direct contact with PSCs induces basement membrane destruction and stromal invasion of organoids via matrix metalloproteinase (MMP) 2, which binds to membrane type-1 MMP (MT1MMP) on PSCs [39]. Öhlund et al. succeeded in the co-culture of pancreatic cancer organoids and fibroblasts and observed their cooperative interactions in terms of growth morphology. The co-cultures revealed another distinct subpopulation of cancer-associated fibroblasts (CAFs) that were located more distantly from neoplastic cells, which provided direct evidence for CAF heterogeneity in pancreatic tumors [62]. In 2021, Koikawa et al. found that the unique prolyl isomerase Pin1 was overexpressed in both cancer cells and CAFs, which was correlated with poor survival in patients with PDAC. They suggested that Pin1 inhibition disrupts the desmoplastic and immunosuppressive tumor microenvironment by acting on CAFs and causing lysosomal degradation of the PD-1 ligand PD-L1 and the gemcitabine transporter ENT1 in cancer cells, rendering pancreatic cancer eradicable by immunochemotherapy [63].

## 7. Personalized Medicine

Personalized medicine is a therapeutic approach based on a patient’s genomic profile, environmental conditions, and lifestyle. Ideally, personalized cancer treatment should be guided by the genomic profile of the patient’s tumor. Based on their properties, organoids established from resected tumors of patients are conceivably very useful in designing personalized medicine because they reflect in vivo properties of the primary tumor and allow in vitro biochemical testing. However, there are limited reports on the successful establishment of a personalized medicine system that combines comprehensive genotyping and organoid culture.

Ideally, personalized cancer treatment should be guided by the genomic profile of the tumor [64,65]. It would be reasonable and efficient to identify genotype-oriented targets and select candidate-targeted drugs for tumors. Alternatively, it may be valid to perform multidrug screening on tumor-derived cell lines to confirm this effect. However, cancer cell lines do not always phenotypically recapitulate human tumors, and the establishment of 2D cancer cell lines from primary tumor materials is very inefficient. The organoid, alternatively called “a miniature of organ”, inherits the phenotype and gene mutations of a primary tumor. According to previous reports, there is little genomic change even after the long-term culture of organoids [37]. Therefore, organoids derived and cultured from fresh tumor samples are expected to be useful in designing personalized medicine. PDCOs have great potential as cancer models for translational research (Table 1).

There are some major applications of the PDCO currently reported as a model for personalized medicine, which is expected to contribute to patients. The first is multi-drug screening, such as high-throughput screening. Organoids are suitable for multi-drug screening because many clones can be cultured in a short period if the culture conditions are adjusted [37,42,58,66,67,68,69,70,71,72,73]. High-throughput screening is legitimate for identifying some effective drugs in a short time or discovering new agents. Saito et al. conducted multi-drug screening of 339 clinically used agents on biliary tract cancer organoids and experimentally demonstrated that 22 compounds suppressed organoid growth, including antifungal agents, HMG-CoA reductase inhibitors, and dopamine D2 receptor agonists [37]. Thus, multi-drug screening, such as high-throughput screening, has the advantage of discovering unexpected results and new drugs. Narasimhan et al. established an organoid from peritoneal disseminated colorectal cancer and performed multi-drug screenings. From the results of drug screening, they were able to find a new agent that was clinically effective in patients with progressive disease after standard surgery and five rounds of chemoradiotherapy [69]. Jiang et al. conducted high-throughput screening of therapeutics in various cancer organoids, which showed that patient-derived organoids recapitulate 97% of gene mutations in their parental tumors, and the drug sensitivity and response matched with more than 80% accuracy between organoids and parental tumors in 21 patients [72].

There are some reports of screening only FDA-approved drugs or clinically available agents for treatment because it is impractical to screen compounds that cannot be used clinically [58,74,75,76,77]. Ramamoorthy et al. developed a multicellular lung organoid called primitive lung-in-a-dish (PLiD), which mimics the lung microstructure with air sac-like alveoli and produces lung surfactant protein. Furthermore, they created lung metastatic cancer models called metastatic tumor-in-a-dish (mTiD), which resembles the architecture of metastatic tumors in the lung with the induction of angiogenesis using PLiD and cancer cells. Finally, they tested the sensitivity of primary and established cancer cells to currently available chemotherapeutic agents and an anti-VEGF antibody in mTiD, which indicated the response of primary patient-derived colon and ovarian tumor cells to therapy in mTiD matched the clinical response of the patient in the clinic [74].

Driehuis et al. examined the correlations of gemcitabine sensitivity between patients’ primary pancreatic cancer tumors and organoids established from them in four patients. The sensitivities to gemcitabine were almost concordant between primary tumors and organoids in all four cases, suggesting that organoids are useful as an in vitro testing model for personalized medicine [58]. Bi et al. successfully established gynecologic cancer organoids from a patient treated prior to surgery with neoadjuvant trastuzumab, which predicted the response to adjuvant chemotherapy and trastuzumab resistance. In addition, the organoid drug sensitivity assay identified alternative treatment options that are currently used in the second-line setting [78].

**Figure 2:**
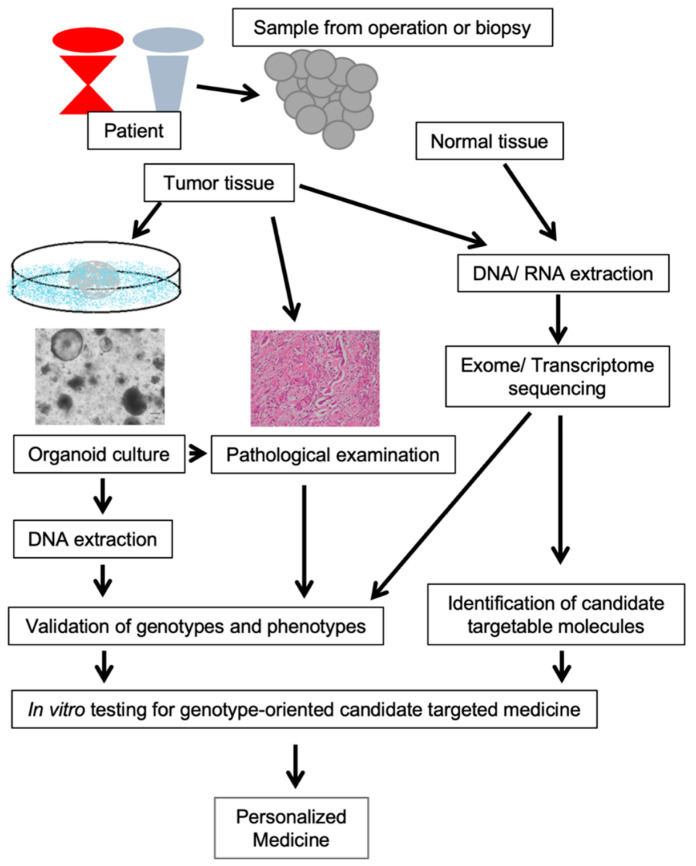
The genotype-oriented personalized medicine model we advocate combining comprehensive genotyping and organoid cultures. By combining exome sequencing and organoid culture, our model enabled the identification of genotype-oriented targets for personalized medicine and tested the efficacy of candidate targeted drugs in organoids.

Further, verification of genome-driven targeted therapies with organoids is very useful for selecting personalized medicine for individual patients [36,41,43,58,79,80,81]. Broutier et al. validated genomic mutations in primary tumors and patient-derived cancer organoids and classified them according to specific molecular pathways. Candidate targeted drugs focusing on these pathways were selected, and their effects were verified using organoids [36]. Driehuis et al. demonstrated that the PRMT5 inhibitor EZP015556, which targets *MTAP*-negative tumors, was effective for a subset of MTAP-positive tumors [58]. Gilles et al. selected targeted therapeutics based on RNA analysis results. The miRNA profiling was performed to determine the status of miRNA deregulation in patients with pancreatic ductal adenocarcinoma. As a validation for the preclinical strategy, the therapeutic potential of a nano-drug, TPN-21, was shown to decrease tumor cell growth and survival in organoids from individual patients [79]. Tung et al. established a model that combines transcriptomic and chromatin profiling. Patient-derived colorectal cancer organoids demonstrated that resistant tumor cells undergo significant chromatin changes in response to oxaliplatin treatment. Integrated transcriptomic and chromatin accessibility analyses using ATAC-Seq and RNA-Seq identified a group of genes associated with significantly increased chromatin accessibility and up-regulated gene expression [81]. We identified genotype-oriented candidate targeted drugs from exome sequencing and tested their efficacy in patients’ cancer organoids derived from patients. Exome sequencing revealed mutations associated with aberrations in signaling pathways, which uncovered candidate targets for targeted therapies. We demonstrated that integrin-linked kinase (ILK) is one such candidate target, and an ILK inhibitor suppressed the proliferation of patient-derived gallbladder cancer organoids. Furthermore, the expression of phosphorylated AKT, the main substrate of ILK, was inversely correlated with the concentration of ILK inhibitors. Our results indicate that organoid culture and exome sequencing are useful for identifying targeted driver mutations and evaluating the efficacy of potential targeted drugs. Our current proof-of-concept approach could increase the therapeutic opportunities for patients with pancreatobiliary cancers [43] (Figure 2).

There have also been some studies on immunotherapy using organoids [82,83,84,85,86,87]. Neal et al. co-cultured primary tumor epithelial cells with endogenous syngeneic tumor-infiltrating lymphocytes (TILs) as a cohesive unit. Their human and murine organoids successfully modeled immune checkpoint blockade with anti-PD-1 and/or anti-PD-L1 expanding and activating tumor antigen-specific TILs and eliciting tumor cytotoxicity [82]. Schnalzger et al. developed a platform to study the cytotoxicity of chimeric antigen receptor (CAR)-engineered lymphocytes in patient-derived colon organoids. They demonstrated efficient targeting in diverse organoids using CAR-engineered NK-92 cells directed toward a ubiquitous epithelial antigen, and tumor antigen-specific cytotoxicity was studied using CAR-NK-92 cells targeting organoids expressing EGFRvIII. Finally, they reported a sensitive in vitro platform to evaluate CAR efficacy and tumor specificity in a personalized manner [83]. Forsythe et al. studied the efficacy of pembrolizumab, ipilimumab, and nivolumab in patient-derived appendiceal cancer organoids. They demonstrated the function of the immune system by culturing organoids with patient-matched immune components derived from blood or lymph nodes/spleen [85]. Gong et al. established a co-culturing platform for organoids and T-cells. In combination with a hydrophobic substrate, they demonstrated that an acoustic droplet printer can yield a large number of homogeneous and highly viable bladder tumor organoids in vitro within a week. By co-culturing these tumor organoids with autologous immune cells, tumor-reactive T cells were induced in vitro. Furthermore, it has also been demonstrated that these tumor-reactive T cells enhance the killing efficiency of matched organoids [86]. Kholosy et al. reported the procedure of a co-culturing system and drug assay of pediatric aggressive malignancies such as diffuse intrinsic pontine glioma or neuroblastoma organoids with immune cells [87].

Although the number of reports is still quite small, other personalized medical models using organoids have been reported. Forsythe et al. used organoids derived from appendiceal cancer or colorectal cancer in 23 cases to verify the effective treatment temperature and drug concentration for hyperthermic intraperitoneal chemotherapy (HIPEC). They concluded that the optimal perfusion protocol varied among patients, and that organoid technology might offer a platform for tailoring HIPEC conditions to the individual patient level [88]. Park et al. prospectively conducted a co-clinical trial with rectal cancer patients and matched patient-derived rectal organoids to determine whether a correlation exists between the experimental results obtained after irradiation in patients and the organoids in the 33 enrolled patients. Their results suggested that radiation responses in patients were positively correlated with those in patient-derived organoids and that the radiosensitivity model could lead to more advanced precision medicine for patients with rectal cancer [89]. Ding et al. suggested the potential of organoids as preclinical models for chemoradiation therapy for nasopharyngeal cancer [90].

There are some challenges to overcome for the clinical application of personalized medicine with patient-derived cancer organoids. First, shortening the culture time and increasing the success rate of organoids are mandatory as follows: Growth rates and culture success rates of organoids vary widely depending on each tumor. Votanopoulos et al. reported that the average turnaround time from organoid development to chemotherapy testing was eight days for appendiceal cancer [91]. In contrast, we found that organoids derived from low-grade bile duct tumors needed a few months to grow for cell viability assays [43]. Furthermore, as mentioned above, the success rate of culture is still low in some tumors such as PBC. The second point is ingenuity for culturing from the following small amount sample: If cancer organoids can be cultured from the specimens from a needle biopsy or liquid cytology, they can be applied to test drugs for an unresectable tumor or even for a resectable tumor for neoadjuvant chemotherapy. There have been some, but still few, reports on such an approach so far [29,92]. The third point is the influence of tumor heterogeneity, which is inevitably a part of the tumor, but does not reflect the entire nature of the tumor itself. A tumor is usually a mass of heterogenic clones, even originating from a single-cell clone; the organoids must have only inherited some parts of the characteristics of the tumor [44]. Fourth, the candidate-targeted therapy approach by genomic profiling may result in no clinically available therapeutic medicine, and high-throughput multi-drug screening including non-anticancer agents could uncover unexpected drugs to be effective for some cancer cells of unique phenotype as mentioned above, which may lead to the development of new antitumor drugs. Furthermore, although the number of cases is still limited, studies on radiation therapy with patient-derived organoids could provide alternative options [93].

## 8. Conclusions

We reviewed the technical development of cancer organoids and their current applications in cancer research and personalized medicine. PDCOs have great potential in the investigation of cancer biology and translational medicine. In particular, it is efficient for testing candidate targeted drugs identified by multi-omics analyses. Currently, studies using organoids are applied not only to targeted drug screening but also to immunotherapy and radiation therapy. Furthermore, platforms constructed by co-culturing cancer organoids with immune cells and stromal cells, such as lymphocytes and fibroblasts, respectively, have been developed, which enable the investigation of the tumor microenvironment using organoids in vitro. There are still some limitations to overcome for clinical applications, such as simplification, speed, and stabilization of culturing and analyzing systems, which can be applied to any cancer. It is hoped that organoids will become more versatile in many tumors and contribute more to cancer research.

## Figures and Tables

**Table 1:** Previous reports of personalized medicine model combined patients-derived cancer organoids.

No	Author	Year	Precision Medicine	Organ
1	Pauli C, et al.	2017	Drug screening (High-throughput)	Various Cancer
2	Broutier L, et al.	2018	Drug screening (Genome-editing target therapy)	Liver
3	Sachs N, et al.	2018	Drug screening (Genome-editing target therapy)	Breast
4	Gilles ME, et al.	2018	Drug screening (Genome-editing target therapy)	Pancreas
5	Mazzocchi AR, et al.	2019	Drug screening (Genome-editing target therapy)	Mesothelioma
6	Hou S, et al.	2019	Drug screening (High-throughput)	Pancreas
7	Saito Y, et al.	2019	Drug screening (High-throughput)	Biliary
8	Kim M, et al.	2019	Drug screening (High-throughput)	Lung
9	Votanopoulos KI, et al.	2019	Drug screening	Appendiceal
10	Kijima T, et al.	2019	Drug screening	Oropharyngeal and Esophageal
11	Neal JT, et al.	2019	Immunotherapy	Melanoma, Renal Cell
12	Ramamoorthy P, et al.	2019	Drug screening (Clinical available drugs)	Lung
13	Li L, et al.	2019	Drug screening	Lung
14	Grassi L, et al.	2019	Drug screening	Renal Cell
15	Schnalzger TE, et al.	2019	Immunotherapy	Colorectal
16	Driehuis E, et al.	2019	Drug screening	Oral
17	Ubink I, et al.	2019	Drug screening (HIPEC)	Colon
18	Tung KL, et al.	2019	Drug screening (Genome-editing target therapy)	Colon
19	Driehuis E, et al.	2019	Drug screening (multiple)	Pancreas
20	Jacob F, et al.	2020	Immunotherapy	Glioblastoma
21	Maenhoudt N, et al.	2020	Drug screening (High-throughput)	Ovary
22	Narasimhan V, et al.	2020	Drug screening (High-throughput)	Colorectal
23	Forsythe SD, et al.	2020	Drug screening (HIPEC)	Colon, Appndiceal
24	Chen JH, et al.	2020	Drug screening (High-throughput)	Lung
25	Shiihara, et al.	2021	Drug screening (Genome-editing target therapy)	Pancreato-Biliary
26	Li Z, et al.	2021	Drug screening (High-throughput)	Lung
27	Luo X, et al.	2021	Drug screening	Colon
28	Jiang S, et al.	2021	Drug screening (High-throughput)	Various Cancer
29	Chen D, et al.	2021	Drug screening	Thyroid
30	Bie Y, et al.	2021	Drug screening (Genome-editing target therapy)	Lung
31	Park M, et al.	2021	Radiation treatment	Rectal
32	Ding RB, et al.	2021	Drug screening, Chemoradiation	Nasopharyngeal
33	Bi J, et al.	2021	Drug screening	Gynecologic
34	Forsythe SD, et al.	2021	Immunotherapy	Appendiceal
35	Larsen BM, et al.	2021	Drug screening (High-throughput)	Various Cancer
36	Bi J, et al.	2021	Drug screening	Endometrial
37	Maier CF, et al.	2021	Drug screening (Clinical available drugs)	Biliary
38	Kazama A, et al.	2021	Drug screening	Renal Cell
39	Kryeziu K, et al.	2021	Drug screening	Rectal
40	Gong Z, et al.	2021	Immunotherapy	Bladder
41	M Kholosy W, et al.	2021	Immunotherapy	Neuroblastoma
42	Boos SL, et al.	2022	Drug screening	Colorectal
43	Dong Y, et al.	2022	Drug screening	Hypopharyngeal
44	Grossman JE, et al.	2022	Drug screening	Pancreas
45	Cho YW, et al.	2022	Drug screening (Clinical available drugs)	Colorectal
46	Reed MR, et al.	2022	Drug screening	Glioblastoma
47	Yuan B, et al.	2022	Drug screening (Clinical available drugs)	Gallbladder
